# Inhalant abuse among youth: the need for urgent intervention to address whitener addiction

**DOI:** 10.11604/pamj.2023.44.156.39315

**Published:** 2023-03-31

**Authors:** Mayur Wanjari, Sampada Late

**Affiliations:** 1 Department of Research and Development, Jawaharlal Nehru Medical College, Datta Meghe Institute of Medical Sciences, Sawangi, Wardha, Maharashtra, India,; 2 Department of Nursing, Government Hospital Samudrapur, Wardha, Maharashtra, India

**Keywords:** Whitener addiction stress, anxiety, depression

## To the editors of the Pan African Medical Journal

We are writing to express our concerns about the alarming rise of inhalant abuse, specifically the growing whitener addiction among youth. As healthcare professionals working in this field, we have seen first-hand the devastating impact of this addiction on young people, their families, and communities worldwide.

Whitener addiction is a form of inhalant abuse that involves the intentional inhalation of fumes from products such as correction fluid, glue, and other solvents. This practice has become increasingly common among young people, who often start using these products to cope with stress, anxiety, or depression [1,2].

The dangers of whitener addiction cannot be overstated. Inhalant abuse can cause various physical and mental health problems, including nausea, dizziness, headaches, seizures, and sudden death. Long-term use can lead to serious health issues, including damage to the brain, liver, and other organs and irreversible neurological damage [3].

The problem of whitener addiction is not limited to one country or region. It is a global health problem that demands urgent attention and action. We need to take a proactive approach to prevent this addiction from spreading and ensure that those affected by it have access to the necessary treatment and support [4,5]. First, we need to increase awareness of the risks and dangers of inhalant abuse, including whitener addiction. Parents, teachers, and healthcare professionals must be educated about the warning signs of inhalant abuse and how to address this problem with young people.

In addition, we need to develop and implement effective prevention and intervention programs to help young people at risk of inhalant abuse. These programs should focus on education, counseling, and support and should be tailored to the needs of each individual.

Finally, we need to invest in research to understand better the causes and consequences of inhalant abuse, including whitener addiction, so that we can develop more effective strategies to prevent and treat this problem.

**Conclusion:** in conclusion, whitener addiction is a serious health problem that is affecting young people around the world. We need to take urgent action to address this problem and to ensure that young people have the knowledge, resources, and support they need to make healthy choices and avoid the dangers of inhalant abuse.
